# Imaging Striatal Microglial Activation in Patients with Parkinson’s Disease

**DOI:** 10.1371/journal.pone.0138721

**Published:** 2015-09-18

**Authors:** Yuko Koshimori, Ji-Hyun Ko, Romina Mizrahi, Pablo Rusjan, Rostom Mabrouk, Mark F. Jacobs, Leigh Christopher, Clement Hamani, Anthony E. Lang, Alan A. Wilson, Sylvain Houle, Antonio P. Strafella

**Affiliations:** 1 Division of Brain, Imaging and Behaviour—Systems Neuroscience, Toronto Western Research Institute, University Hospital Network, University of Toronto, Toronto, Ontario, Canada; 2 Research Imaging Centre, Centre for Addiction and Mental Health, University of Toronto, Toronto, Ontario, Canada; 3 Department of Human Anatomy and Cell Science, Faculty of Health Sciences, University of Manitoba, Winnipeg, Manitoba, Canada; 4 Division of Neurosurgery, Toronto Western Hospital, University Hospital Network, University of Toronto, Toronto, Ontario, Canada; 5 Morton and Gloria Shulman Movement Disorder Unit & Edmond.J. Safra Program in Parkinson Disease, Toronto Western Hospital, University Hospital Network, University of Toronto, Toronto, Ontario, Canada; Emory University, UNITED STATES

## Abstract

This study investigated whether the second-generation translocator protein 18kDa (TSPO) radioligand, [^18^F]-FEPPA, could be used in neurodegenerative parkinsonian disorders as a biomarker for detecting neuroinflammation in the striatum. Neuroinflammation has been implicated as a potential mechanism for the progression of Parkinson’s disease (PD). Positron Emission Tomography (PET) radioligand targeting for TSPO allows for the quantification of neuroinflammation *in vivo*. Based on genotype of the rs6791 polymorphism in the TSPO gene, 16 mixed-affinity binders (MABs) (8 PD and age-matched 8 healthy controls (HCs)), 16 high-affinity binders (HABs) (8 PD and age-matched 8 HCs) and 4 low-affinity binders (LABs) (3 PD and 1 HCs) were identified. Total distribution volume (V_T_) values in the striatum were derived from a two-tissue compartment model with arterial plasma as an input function. There was a significant main effect of genotype on [^18^F]-FEPPA V_T_ values in the caudate nucleus (*p* = 0.001) and putamen (*p* < 0.001), but no main effect of disease or disease x genotype interaction in either ROI. In the HAB group, the percentage difference between PD and HC was 16% in both caudate nucleus and putamen; in the MAB group, it was -8% and 3%, respectively. While this PET study showed no evidence of increased striatal TSPO expression in PD patients, the current findings provide some insights on the possible interactions between rs6791 polymorphism and neuroinflammation in PD.

## Introduction

Neuroinflammation is considered to play an important role in the progression of Parkinson’s disease (PD). Neuroinflammation in PD was first evidenced in a postmortem study where activated microglia were found in the substantia nigra (SN) [1]. A subsequent postmortem study further identified activated microglia in the extended brain areas such as putamen, hippocampus, as well as trans-entorhinal, cingulate, and temporal cortices [2]. Neuroinflammatory processes were also confirmed by increased concentration of inflammatory cytokines such tumor necrosis factor α (TNFα) and interleukins 1β and 6 in the striatum at postmortem [3] as well as in *vivo* studies using the serum [4] and cerebrospinal fluid [5] of PD patients.

Translocator protein 18 kDa (TSPO) has been studied as a potential *in vivo* biomarker of reactive gliosis and inflammation associated with a variety of neuropathological conditions [6]. TSPO is located in the outer mitochondrial membrane of glial cells. While TSPO levels are very low in healthy brains, they markedly increase co-localizing activated microglia in brains affected by various diseases such as amyotrophic lateral sclerosis, Alzheimer disease, frontotemporal dementia and multiple sclerosis [7]. This elevated TSPO expression was primarily quantified using [^11^C](*R*)PK11195 positron emission tomography (PET), the first and most commonly used TSPO radioligand.

To date, a few studies have investigated neuroinflammation in PD patients using [^11^C](*R*)PK11195 PET. However, the findings have been inconclusive. While some studies have found elevated TSPO binding in the nigro-striatal regions [8], others did not support these observations [9]. Additionally, an association between elevated TSPO binding in the nigro-striatal regions and clinical measures has been demonstrated in some studies [8] but not in others [9]. One possible explanation for these inconsistent findings may be due to the limitations inherent to this prototypical radioligand ([^11^C](*R*)PK11195) such as low signal-to-noise ratio, high non-specific binding, low brain penetration, and high plasma protein binding. These limitations as well as its limited dissemination for wider clinical use due to the short-lived carbon-11 labelling have prompted the development of new TSPO radioligands.

[^18^F]-FEPPA is a new, second-generation TSPO radioligand that has shown a high affinity for TSPO, an appropriate metabolic profile, high brain penetration and good pharmacokinetics [10]. Its quantification has been validated using the two-tissue compartment model with total distribution volume (V_T_) as reliable outcome measure [11]. [^18^F]-FEPPA demonstrated superior specificity to the affected side of the striatum and stronger correlation with inflammatory cytokines compared with [^11^C](*R*)PK11195 using an animal model of PD [12]. Further, our group recently demonstrated a three-fold higher [^18^F]-FEPPA V_T_ in a tumor site compared to the healthy contralateral area in an individual subject [13], as well as a significant increase in individuals with major depressive episode [14] and patients with Alzheimer’s disease (AD) [15].

Second-generation TSPO radioligands have been known to present three patterns of binding affinity based on genetic polymorphism: high-affinity binders (HABs), mixed-affinity binders (MABs), and low-affinity binders (LABs) [16]. These different phenotype patterns can be predicted by a single-nucleotide polymorphism (SNP), rs6971 located in the exon 4 of the TSPO gene resulting in a nonconservative amino-acid substitution at position 147 from alanine to threonine (Ala147Thr) in the fifth transmembrane domain of the TSPO protein. This polymorphism accounts for some of the large inter-individual variability in the outcome measures [16]. Therefore, accounting for this polymorphism in the statistical analyses will likely increase sensitivity in detecting neuropathological changes [17].

In the present study, we investigated for the first time whether TSPO imaging with [^18^F]-FEPPA, could be used as a potential biomarker for neuroinflammation in PD. Using the striatum as our main region of interest (ROI), we sought to determine whether (1) the polymorphisms would reflect on TSPO binding affinity also in PD patients; (2) [^18^F]-FEPPA V_T_ could differentiate the PD group from the healthy control (HC) group; and (3) the levels of TSPO binding correlate with clinical measures of PD.

## Materials and Methods

### Subjects

Nineteen patients meeting UK Brain Bank criteria for the diagnosis of idiopathic PD and 17 HC subjects participated in the study. Exclusion criteria for all participants included (1) history of a head injury, psychiatric or neurological (except PD for the patients) diseases, (2) Alcohol or drug dependency or abuse, (3) contraindications for MRI scanning, and (4) use of nonsteroidal anti-inflammatory drugs. PD patients were assessed for their cognitive ability using the Montreal Cognitive Assessment (MoCA), the level of depression using the Beck Depression Inventory II, as well as for motor severity of the disease using the motor subset of the Unified Parkinson Disease Rating Scale (UPDRS-III). All participants underwent PET and structural MRI scans. All participants provided written informed consent following full explanation of the study procedures. The study was approved by the Centre for Addiction and Mental Health Research Ethics Board and the University Health Network Research Ethics Board, and conformed to the *Declaration of Helsinke*.

### PET data acquisition

The synthesis of [^18^F]-FEPPA has been described in detail elsewhere [10]. It can be reliably and quickly labeled with [^18^F] by nucleophilic displacement of a tosylate, leaving group in a fast one-step reaction yielding a sterile and pyrogen-free product after purification and formulation.

The PET images were obtained using a 3D High Resolution Research Tomography (HRRT) (CPS/Siemens, Knoxville, TN, USA), which measures radioactivity in 207 slices with an inter-slice distance of 1.22 mm. A custom-fitted thermoplastic mask was made for each subject and used with a head fixation system during the PET scans to minimize head movement. Following a transmission scan, intravenous [^18^F]-FEPPA was administered as a bolus. The mean injected amount, specific activity at the time of injection and mass injected of all the participants were 4.926 ± 0.265 (mCi), 3127.723 ± 2026.481 (mCi/μmol), and 0.9 ± 0.676 (μg), respectively. The scan duration was 125 min. The images were reconstructed into 34 time frames: 1 frame of variable length until the radioactivity appears in the field of view (FOV), 5 frames of 30s, 1 frame of 45s, 2 frames of 60s, 1 frame of 90s, 1 frame of 120s, 1 frame of 210s, and 22 frames of 300s.

All PET images were corrected for attenuation using a single photon point source, ^137^Cs (T_50_ = 30.2 years, Eγ = 662 keV) and were reconstructed by filtered back projection algorithm using a HANN filter at Nyquist cutoff frequency. The reconstructed image has 256 × 256 × 207 cubic voxels measuring 1.22 × 1.22 × 1.22 mm^3^ and the resulting reconstructed resolution is close to isotropic 4.4 mm, full width at half maximum in plane and 4.5 mm full width at half maximum axially, averaged over measurements from the center of the transaxial FOV to 10 cm off-center in 1.0 cm increments. In addition, for frame realignment for head motion correction, each image was reconstructed without attenuation correction using three iterations of iterative reconstruction [11].

### MRI acquisition

MRI images for all the subjects were acquired for co-registration with the corresponding PET images and the anatomical delineation of the ROIs (i.e., caudate and putamen). Proton density (PD)-weighted MR images were chosen for better identification of the striatum [18]. 2D oblique PD-weighted MR images were acquired with a General Electric Discovery 3.0 T MRI scanner (slice thickness = 2 mm, repetition time (TR) = 6000 ms, echo time (TE) = Min Full, flip angle = 90°, number of excitations (NEX) = 2, acquisition matrix = 256 × 192, and Field of View = 22 cm).

### Input function measurement

Dispersion- and metabolite-corrected plasma input function was generated as previously described [11]. Briefly, arterial blood was taken continuously at a rate 2.5 mL/min for the first 22.5 minutes after radioligand injection and the blood radioactivity levels were measured using an automatic blood sampling system (Model # PBS-101 from Veenstra Instruments, Joure, The Netherlands). In addition, 4 to 8 ml manual arterial blood samples were obtained at 2.5, 7, 12, 15, 30, 45, 60, 90, and 120 min relative to time of injection [11]. A bi-exponential function was used to fit the blood-to-plasma ratios. A Hill function was used to fit the percentage of unmetabolized radioligand. The dispersion effect was modeled as to the convolution with a mono-exponential with dispersion coefficient of 16 seconds and corrected with iterative deconvolution [19].

### Generation of ROI-based Time activity curve (TAC)

[^18^F]-FEPPA PET images were preprocessed and ROIs were automatically generated using in-house software, ROMI [16]. Briefly, ROMI fits a standard template of ROIs to an individual PD-weighted MR image based on the probability of gray matter, white matter, and CSF. The individual MR images are then co-registered to each summed [^18^F]-FEPPA PET image using the normalized mutual information algorithm so that individual refined ROI template can be transferred to the PET image space to generate the time activity curve (TAC) for each ROI. Our *a priori* ROIs included the caudate nucleus and putamen, which are disease-affected regions and whose quantification was validated [11]. Dynamical series of images of [^18^F]-FEPPA PET were visually checked for head motion and corrected using frame-by-frame realignment. Low noise, nonattenuation-corrected images (created with iterative reconstruction) were used to optimize the frame-by-frame realignment process. A normalized mutual information algorithm was applied with SPM8 (Wellcome Trust Centre for Neuroimaging, London, UK) to co-register each frame to the frame that showed a high signal-to-noise ratio. Parameters from the normalized mutual information were applied to the corresponding attenuation-corrected dynamic images to generate a movement-corrected dynamic image.

To address the potential issues of bias from the volume loss in the older subjects, time activity data for all subjects was corrected for the effect of partial volume error (PVE) using the Mueller-Gartner partial volume error correction algorithm as implemented in Bencherif et al (2004) [20].

### Kinetic analysis

Total distribution volume (V_T_) values in each ROI were derived from a two-tissue compartment model (2-TCM) using [^18^F]-FEPPA radioactivity in arterial plasma as an input function and a 5% vascular contribution [11]. V_T_ is a ratio at equilibrium of the radioligand concentration in tissue to that in plasma (i.e. specific binding and non-displaceable uptake including non-specifically bound and free radioligand in tissue) and can be expressed in terms of kinetic rate parameters as: V_T_ = K_1_ / k_2_ (1 + k_3_ / k_4_) where K_1_ and k_2_ are influx and efflux rates for radiotracer passage across the blood brain barrier and k_3_ and k_4_ describe the radioligand transfer between the free and non-specific compartments and the specific binding compartment. We also measured the percentage of the coefficient of variation (%*COV* = 100% x standard error/mean), where standard error was estimated from the diagonal of the covariance matrix of nonlinear least-squares fitting [11]. From the different ROIs, we included V_T_ with %*COV* of ≤ 20, which assured less data noise.

### DNA extraction and polymorphism genotyping

Genomic DNA was obtained from peripheral leukocytes using high salt extraction methods [21]. The polymorphism rs6971 was genotyped variously using a TaqMan® assay on demand C_2512465_20 (AppliedBiosystems, CA, USA). The allele T147 was linked to Vic and the allele A147 was linked FAM. PCR reactions were performed in a 96-well microtiter-plate on a GeneAmp PCR System 9700 (Applied Biosystems, CA, USA). After PCR amplification, end point plate read and allele calling was performed using an ABI 7900 HT (Applied Biosystems, CA, USA) and the corresponding SDS software (v2.2.2). Individuals with genotype Ala147/Ala147 were classified as HABs, Ala147/Thr147 as MABs, and Thr147/Thr147 as LABs [16].

### Statistical analysis

Demographic and clinical measures were compared using factorial analysis of variance (ANOVA), independent, two-tailed student *t* tests, or Fisher’s exact tests. Group differences in V_T_ values were analyzed using factorial ANOVA with TSPO genotype and disease as fixed factors in the caudate nucleus and the putamen. A second level of analysis with student *t* tests were performed among four groups: (1) between HC-MAB and HC-HAB, (2) between PD-MAB and PD-HAB, (3) between HC-MAB and PD-MAB, and (4) between HC-HAB and PD-HAB. Correlations were investigated between clinical measures and V_T_ values in the ROIs using Pearson’s correlation tests. All of the statistical analyses were performed using SPSS Statistics version 20.0. The threshold for significance was set at *p* < 0.05.

## Results

### Demographic and clinical characteristics

Based on the rs6971 polymorphism, there were 16 MABs consisting of 8 HC subjects and 8 PD patients, 16 HABs consisting of 8 HC subjects and 8 PD patients, as well as 4 LABs consisting of 3 PD patients and 1 HC. [^18^F]-FEPPA is not quantifiable in these low affinity binders and for this reason these subjects were not included in the analyses. In general, LAB subjects in a Caucasian sample represent less than 5% [17], and only very few are identified in any given study.

The factorial ANOVA showed that there were no significant differences in age (*F*_(3, 28)_ = 0.859, *p* = 0.474), as well as in the [^18^F]-FEPPA injected amount (*F*_(3, 28)_ = 1.296, *p* = 0.295), specific activity at the time of injection (*F*_(3, 28)_ = 0.939, *p* = 0.435) or mass injected (*F*_(3, 28)_ = 1.679, *p* = 0.194) between MABs and HABs or between HC and PD groups. Fisher’s exact tests showed that there were no significant differences in the composition of gender and handedness in the four groups or in the composition of symptom dominant side between PD-MAB and PD-HAB groups (*p* > 0.05). In addition, the PD-MAB and PD-HAB groups were not significantly different in years of education, MoCA scores, BDI scores, UPDS III, duration of disease, or total LEED (*p* > 0.05) (Table 1).

**Table 1:** Demographic and clinical characteristics of PD patients.

	PD-MAB (n = 8)	PD-HAB (n = 8)
**Age**	67.1 (10.8)	61.5 (6.2)
**Gender (M:F)**	7:1	4:4
**Handedness (R:L)**	8:0	8:0
**Education**	15.5 (3.8)	16.4 (3.2)
**MoCA**	27.4 (2.3)	26.5 (1.9)
**BDI**	6.4 (4.1)	7.6 (4.3)
**Symptom dominant side (R:L)**	6:2	4:4
**UPDRS III**	28.4 (9.6)	25.0 (5.1)
**Duration of disease**	5.6 (2.1)	5.9 (4.7)
**Total LEDD**	623.8 (596.4)	525.1 (267.6)

UPDRS-III, Unified Parkinson’s Disease Rating Scale III; MoCA, Montreal Cognitive Assessment; BDI, Beck Depression Inventory II; LEDD, Levodopa Equivalent Daily Dose.

### Genotype and disease effects on TSPO binding

Levene’s tests of equality of error variances were not significant in either caudate nucleus (*p* = 0.449) or putamen (*p* = 0.284). There was a significant main effect of genotype on V_T_ values in the caudate nucleus (*F*_(1, 28)_ = 14.62, *p* = 0.001) and in the putamen (*F*_(1, 28)_ = 27.15, *p* < 0.001). There was no main effect of disease in the caudate nucleus (*F*_(1, 28)_ = 0.37, *p* = 0.55) or in the putamen (*F*_(1, 28)_ = 1.27, *p* = 0.269). There was also no disease x genotype interaction in the caudate nucleus (*F*_(1, 28)_ = 1.38, *p* = 0.25) or in the putamen (*F*_(1, 28)_ = 0.86, *p* = 0.362).

**Fig 1:**
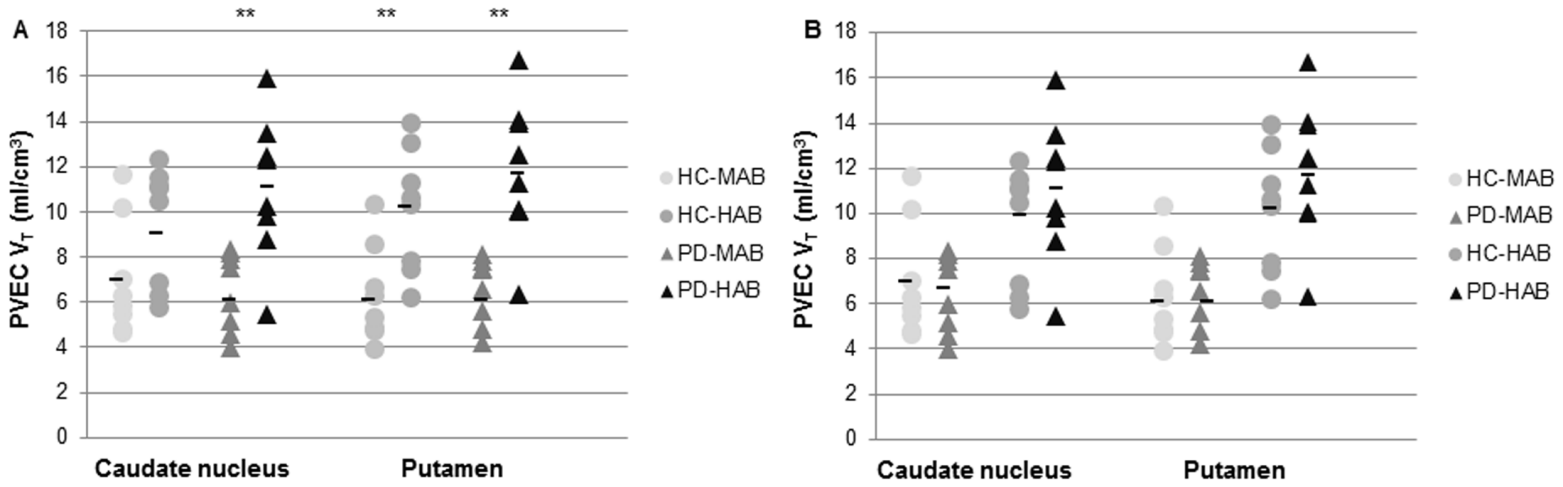
Graphs of partial volume effect corrected (PVEC) total distribution volume (V_T_) in the caudate nucleus and in the putamen. A. Healthy control with mixed affinity binder (HC-MAB) and healthy control with high affinity binder (HC-HAB) groups as well as Parkinson’s disease with mixed affinity binder (PD-MAB) and Parkinson’s disease with high affinity binder (PD-HAB) groups. Asterisks indicate that the HAB groups show significantly higher V_T_ mean values compared with the MAB groups (** *P* < 0.01). B. Healthy control with mixed affinity binder (HC-MAB) and Parkinson’s disease with MAB (PD-MAB) groups as well as healthy control with high affinity binder (HC-HAB) and Parkinson’s disease with high affinity binder (PD-HAB) groups. In the HAB group, the percentage difference between PD and HC was 16% in both caudate nucleus and putamen, and in the MAB group, it was -8% and 3%, respectively.

Using student *t* tests, when comparing the effect of genotype in HC groups, HC-HAB group showed significantly higher V_T_ values in in the putamen (*p* = 0.009), but not in the caudate nucleus (*p* = 0.084) compared with HC-MAB group. Similarly, the effect of genotype in PD groups showed that PD-HAB had significantly higher V_T_ values in both caudate nucleus (*p* = 0.003) and putamen (*p* = 0.001) compared with PD-MAB (Fig 1A). When testing the effect of the disease, neither PD-MAB nor PD-HAB group showed significant increase in V_T_ values in any ROI compared with HC-MAB and HC-HAB groups, respectively (Fig 1B). The percentage differences of the mean V_T_ values between PD-HAB and HC-HAB groups were 16% in both caudate nucleus and putamen, while they were -8% and 3%, respectively in the MAB groups.

There was no correlation between LEDD, UPDRS scores or duration of disease with the V_T_ values in the striatum in either PD-HAB or PD-MAB groups, thus excluding any role of these variables in the V_T_ findings.

**Fig 2:**
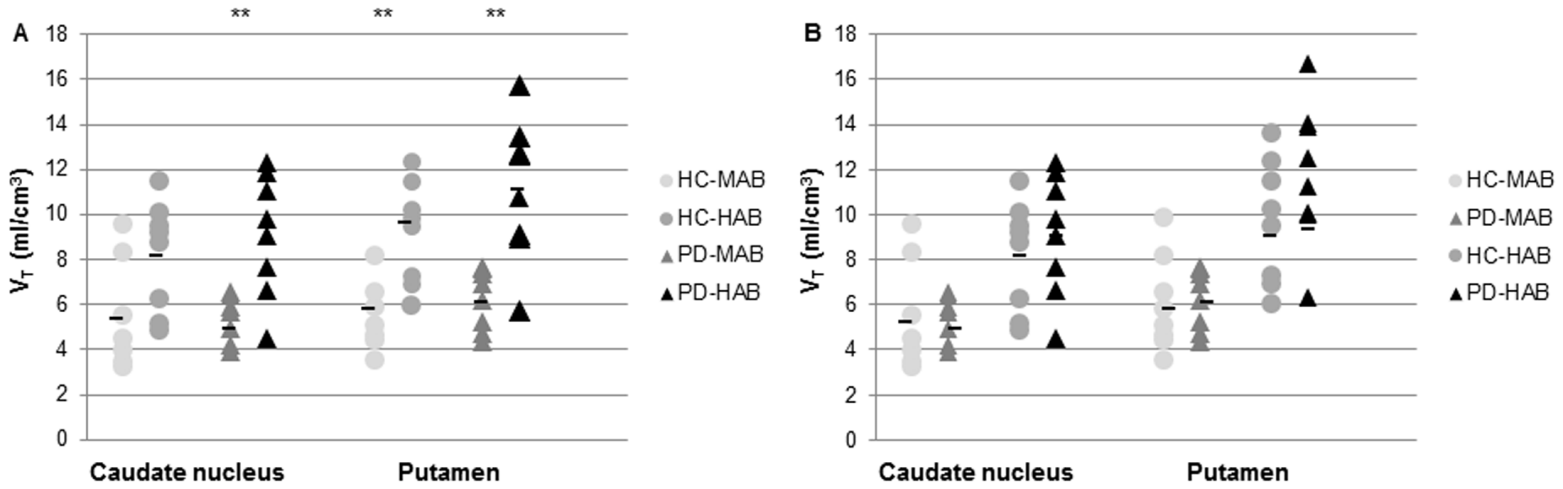
Graphs of total distribution volume (V_T_) in the caudate nucleus and in the putamen. A. Healthy control with mixed affinity binder (HC-MAB) and healthy control with high affinity binder (HC-HAB) groups as well as Parkinson’s disease with mixed affinity binder (PD-MAB) and Parkinson’s disease with high affinity binder (PD-HAB) groups. Asterisks indicate that the HAB groups show significantly higher V_T_ mean values compared with the MAB groups (** *P* < 0.01). B. Healthy control with mixed affinity binder (HC-MAB) and Parkinson’s disease with MAB (PD-MAB) groups as well as healthy control with high affinity binder (HC-HAB) and Parkinson’s disease with high affinity binder (PD-HAB) groups. In the HAB group, the percentage difference between PD and HC was 11% in the caudate nucleus and 14% in the putamen, and in the MAB group, it was -3% and 5%, respectively.

We conducted as well an additional analysis on the data without PVE correction to investigate whether this played any role in the results. The analysis confirmed the main effect of genotype on V_T_ values (Fig 2A), but no main effect of disease (Fig 2B), or disease x genotype interaction.

## Discussion and Conclusions

This is the first study to investigate the potential use of [^18^F]-FEPPA as a new radioligand to measure neuroinflammation in PD patients. As already observed in normal controls, we found that in PD patients as well, the rs6971 polymorphism influenced TSPO binding affinity. Although, we did not find a significant disease effect on TSPO expression in the striatum, an interesting observation was a trend towards elevated TSPO binding in the PD-HAB group (16% in both caudate nucleus and putamen), but not in the PD-MAB group. These observations imply that while the genotype of the rs6791 polymorphism certainly plays a role in TSPO expression also in PD, a larger sample size may be needed to investigate the interactions between rs6791polymorphism and neuroinflammation in PD patients, although other studies with similar sample size did show a significant increase in [^18^F]-FEPPA V_T_ in individuals with major depressive episode [14] and patients with AD [15].

In those studies, such differences in TSPO expression were reported mainly in the HAB groups. In particular in AD patients [15], elevated microglia was detected throughout the brain, in both gray and white matter suggesting that neuroinflammation may certainly play a role in the cognitive decline.

The same dissociation and potential role of the rs6971 polymorphism on TSPO binding has also been suggested in studies using other second-generation TSPO radioligands. For example using [^3^H]PBR28, postmortem brains of patients with schizophrenia classified as HABs showed significantly greater specific binding in the dorsolateral prefrontal cortex while those categorized as MABs did not [22]. Similarly using [^18^F]FEMPA in AD, the disease effect on TSPO expression was stronger in different cerebral regions in HAB patients [23]. On the other hand, cocaine abusers did not seem to demonstrate the same dissociation [24].

The rs6791 polymorphism can be associated with the susceptibility, progression or disease protection [25]. For instance, healthy individuals with Ala147/Ala147 (i.e., HABs) have been associated with significantly higher level of low-density lipoprotein (LDL) than those with Ala147/Thr147 (i.e., MABs) and with Thr147/Thr147 (i.e., LABs) [26], and LDL has been associated with the risk of PD [27]. While these reports are highly suggestive, the clinical significance of this polymorphism in PD is yet to be determined. Similarly, despite the well-documented increase in the TSPO expression as a result of brain insult in *ex-vivo* brain tissue, the functional significance of the upregulated TSPO is still unclear [6]. One possibility is that this upregulation may be related to glial proliferation, migration, and phagocytosis [28] or secretion of inflammatory cytokines. [^11^C](*R*)PK11195 reduced the expression of proinflammatory cytokines in cultured human microglia [29]. Furthermore, increased TSPO levels in microglia and astrocytes may possibly increase neurosteroid synthesis at injury sites to promote neurotropic and neuroprotective activity [30]. While the observations regarding the potential role of the polymorphism on TSPO binding may be quite intriguing, they are speculative and warrant further investigations.

Consistent with previous studies [9], we did not observe any relationship between antiparkinsonian medication, disease severity and duration of disease and [^18^F]-FEPPA V_T_ in the striatum, thus implying that these variables are not associated with TSPO expression in PD patients.

In conclusion, while our findings did not reveal a significant disease effect on [^18^F]-FEPPA V_T_ in the striatum, further investigations are warranted to determine whether [^18^F]-FEPPA could be used as a biomarker of neuroinflammation in PD and to understand the interactions between the rs6791polymorphism, neuroinflammation, and clinical subtypes as well as disease prognosis.
